# *Solobacterium moorei* sepsis secondary to flexor tenosynovitis: a case report and review of literature

**DOI:** 10.1128/asmcr.00054-25

**Published:** 2025-07-08

**Authors:** Katarina Popovic, Satya Sai Venkata Lakshmi Arepalli, Sachin Saju, Douglas Drevets

**Affiliations:** 1 Department of Internal Medicine, University of Oklahoma Health Sciences Center 173793, Oklahoma City, Oklahoma, USA; 2 Section of Infectious Diseases, Department of Medicine, University of Oklahoma Health Sciences Center 173793, Oklahoma City, Oklahoma, USA; Vanderbilt University Medical Center, Nashville, Tennessee, USA

**Keywords:** *Solobacterium moorei*, bacteremia, sepsis, flexor tenosynovitis, MALDI-TOF MS

## Abstract

**Background:**

*Solobacterium moorei* is a gram-positive, non-sporulating, strict anaerobic bacillus and an uncommon human pathogen typically found in skin and soft tissue infections. Additionally, *S. moorei* is a rare cause of severe infections associated with bacteremia.

**Case Summary:**

We report a case of a 56-year-old African American man with *S. moorei* bacteremia, likely due to a bite wound, and review 25 previously reported cases. The patient recovered after incision and drainage of flexor tenosynovitis and treatment with 15 days of aminopenicillin/beta-lactamase inhibitor antibiotics.

**Conclusion:**

*S. moorei* was identified with matrix-assisted laser desorption/ionization time-of-flight mass spectrometry, whereas most other reports used 16s RNA sequencing. Literature review indicates isolates are typically susceptible to penicillins, beta-lactam/beta-lactamase inhibitors, carbapenems, and 3rd/4th-generation cephalosporins but may be resistant to metronidazole, levofloxacin, and rifampin. Improvements in diagnostic methods may lead to more frequent identification of *S. moorei* in cases of severe sepsis.

## INTRODUCTION

*Solobacterium moorei* is the only known species of the *Solobacterium* genus. It is a gram-positive, non-sporulating, strict anaerobic bacillus that is part of the oral and intestinal microbiota. It is a rare human pathogen predominantly causing skin and soft tissue infections. It is a very rare cause of severe infections associated with bacteremia, with only 25 previous cases reported identified by literature search. Here, we report a case of *S. moorei* bacteremia with sepsis secondary to flexor tenosynovitis.

## CASE PRESENTATION

A 56-year-old African American man experiencing homelessness with underlying schizoaffective disorder, polysubstance abuse disorder, and attention deficit hyperactivity disorder (ADHD) presented to the emergency department with severe pain, swelling, and decreased range of motion in his right hand.

**Fig 1:**
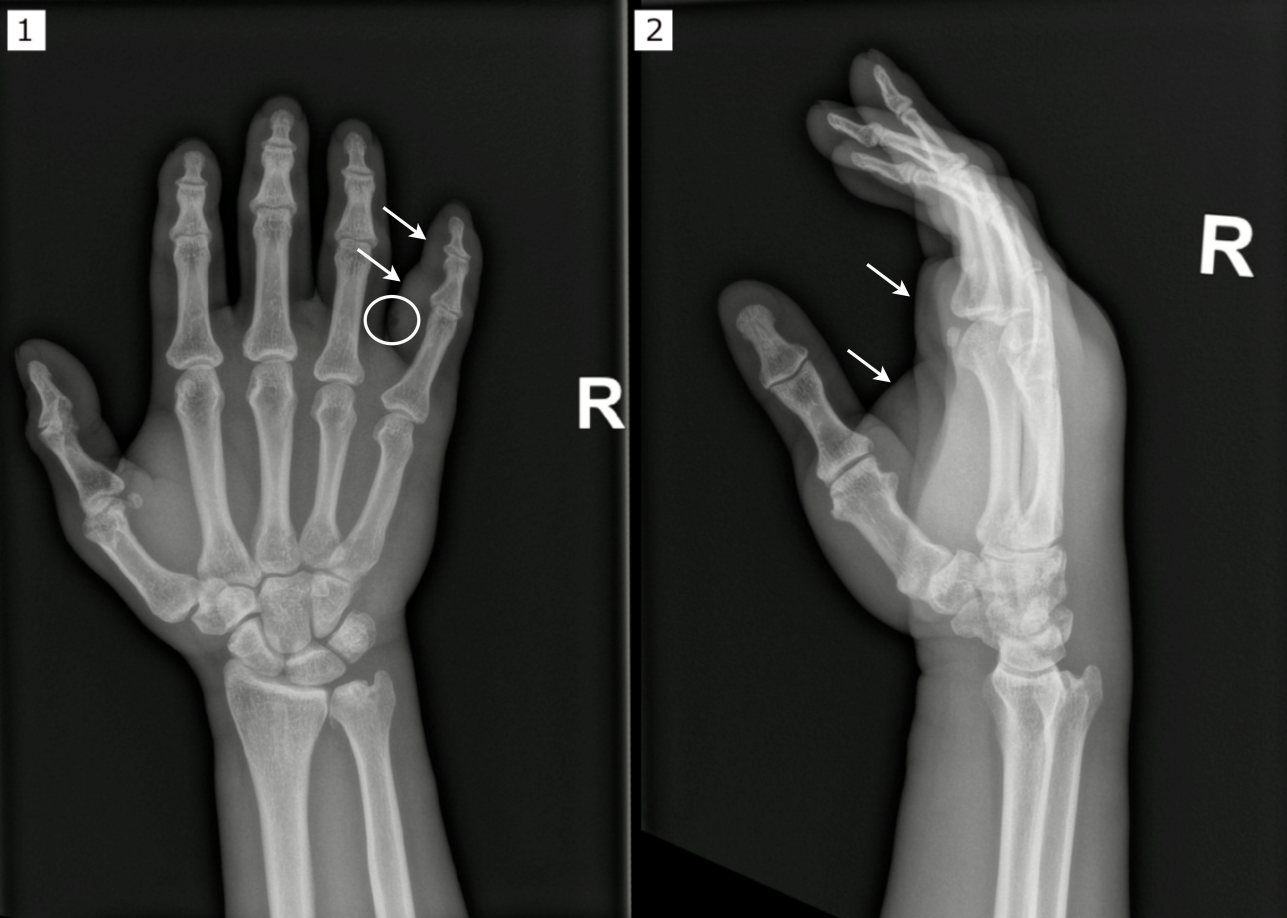
X-ray of the patient’s right hand in (1) posteroanterior and (2) lateral projections with soft tissue swelling and subcutaneous emphysema on the palmar aspect of the fifth proximal phalanx. The circle demonstrates where the subcutaneous emphysema is located, while the arrows indicate soft tissue swelling.

He reported biting off a callus on the palmar side of the proximal phalanx of the right fifth finger about 2 weeks prior to presentation. The following day, he noted swelling and pain in his right hand. He developed fevers, chills, and dizziness a few days prior to seeking medical care. Physical examination revealed significant swelling and erythema of the entire right hand and forearm, most prominent on the fifth digit with a greatly reduced range of motion of each finger on that hand. He was febrile (38.4°C), tachycardic (90–120 bpm), and hypertensive (180–200/100 mmHg), meeting the systemic inflammatory response syndrome criteria for sepsis. Notable laboratory tests included a white blood cell count of 21.31 × 10^3^/µL (normal 4.0–11.0 × 10^3^/µL) with 81.9% neutrophils, lactate of 2.7 mmol/L (normal 0.5–1.9 mmol/L), C-reactive protein 162.2 g/L (normal < 5.0 mg/L), and creatinine of 1.4 mg/dL (normal 0.78–1.34 mg/dL). The urine drug screen was positive for methamphetamine, cocaine, and cannabinoids, whereas human immunodeficiency virus (HIV), hepatitis B, and hepatitis C serologies were non-reactive. An X-ray of his right hand showed soft tissue swelling and subcutaneous emphysema on the palmar aspect of the fifth proximal phalanx (Fig. 1). He was treated with fluid resuscitation, cefepime 2 g every 8 h, and vancomycin (25 mg/kg loading dose with maintenance dosing adjusted by vancomycin trough levels) and underwent emergent incision and drainage (I&D) on admission and on hospital days two and four. During the initial I&D, significant purulence was encountered upon deep dissection of the A1 pulley of the right small finger. Aerobic and anaerobic cultures were collected from the tissue.

Due to a shortage of blood culture bottles, a single blood culture sample was collected using BACTEC Plus Aerobic/F bottle. The instrument bottle was incubated using the BC BACTEC Automated Blood Culture System and subcultured to Columbia blood agar and CDC anaerobic blood agar per the microbiology laboratory standard protocol. All subcultures were incubated at 35–37°C in CO_2_. Bacterial growth was strictly anaerobic with pinpoint colonies. Time to positivity of the blood culture from the moment it was subcultured was roughly 24 h. The Gram stain was initially reported as gram-negative bacilli; however, the Genmark ePlex Blood Culture Nucleic Acid Test (GenMark, Roche Diagnostics USA, Indianapolis, Indiana, USA) for gram-negative bacteria was negative. Matrix-assisted laser desorption/ionization time of flight mass spectrometry (MALDI-TOF MS) (BD Bruker MALDI-TOF MS Microflex, software version flexAnalysis 3.4.79.0) was performed on growth from a subculture and revealed *S. moorei* with an ID/confidence score of 2.49, indicating high confidence. Susceptibility testing was performed using the E-test method (ETEST, bioMérieux, Hazelwood, Missouri, USA) under anaerobic conditions at 35°C for 48 h. The medium used was pre-reduced Brucella blood with vitamin K and Hemin agar and the inoculum in brain heart infusion broth. E-test results were interpreted using the 35th edition Clinical & Laboratory Standards Institute (CLSI) M100 Performance Standards for Antimicrobial Susceptibility Testing for anaerobes (1). Susceptibility testing revealed resistance to metronidazole with a minimal inhibitory concentration (MIC) > 256 µg/mL and susceptibility to piperacillin-tazobactam (MIC 0.064 µg/mL), ampicillin-sulbactam (MIC 0.032 µg/mL), and clindamycin (MIC 0.125 µg/mL). Intraoperative cultures from the initial I&D grew *Streptococcus constellatus*, *Prevotella* species, and *Fusobacterium* species. A second blood culture obtained 3 days after admission was sterile. After susceptibility testing was completed, antibiotics were deescalated on day seven to intravenous ampicillin-sulbactam 3 g every 6 h, and oral amoxicillin-clavulanic acid 875–125 mg every 12 h was prescribed on discharge to complete a 15-day course of antibiotics from source control. Clinical improvement was noted by resolution of fever by day two with decreased swelling and restored range of motion in his fingers.

## DISCUSSION

*S. moorei* is a gram-positive, non-sporulating, strict anaerobe, rod-shaped bacteria and the only species in the genus *Solobacterium*. It was first described in 2000 by Kageyama et al. after isolation from human feces and also found in oral (particularly tongue) and intestinal microbiota. Despite phenotypic resemblance to *Eubacterium* species, phylogenetic analysis with 16s rDNA sequencing revealed it was distinct from *Eubacterium*, *Erysipelothrix rhusiopathiae*, and *Holdemania filiformis*, leading to a new genus: *Solobacterium* (2). It is implicated in halitosis (3, 4) and periodontal and endodontic diseases (5) and may play a role in colorectal carcinogenesis along with other intestinal anaerobes (6).

*S. moorei* is considered an opportunistic pathogen and rarely causes severe infections. Including the current case, there are only 26 reported cases of *S. moorei* bacteremia. These typically arise after damage to mucosal or cutaneous barriers (7). Most patients have serious underlying illnesses, with the most common being malignancies (Table 1) (8–12). Two cases have been reported in patients with intravenous drug abuse (10, 13). One case was associated with acute cholangitis in the setting of chronic pancreatitis and bile duct stricture (14) and one with HIV infection and herpes simplex virus 1 (HSV-1) esophagitis (15). Interestingly, Alejo-Cancho et al. reported a case in a 19 year-old with no significant medical history in the setting of left maxillary sinusitis with subperiosteal abscess (16). In the present case, the patient had a history of polysubstance abuse disorder but denied using drugs intravenously.

**TABLE 1:** Reported cases of bacteremia with *Solobacterium* species^*a*^

No.	Age/sex	Infection	Comorbidities	ID	Other isolated bacteria	Treatment	Treatment duration	Yr (ref.)
1	67 M	Sepsis; multiple dentoalveolar abscesses	Multiple myeloma; h/o autologous bone marrow graft	16s rRNA PCR	None	FEP; source control: dental extraction	15 days	2006 (8)
2	43 F	Acute proctitis post-radiotherapy	Cervical cancer	16s rRNA PCR	None	TZP	14 days	2006 (9)
3	37 M	Septic pulmonary embolism; femoral vein thrombophlebitis	Intravenous drug abuse	16s rRNA PCR	*Fusobacterium nucleatum; Bacteroides ureolyticus*	PCNG + FLU → CLI → PCNG + MTZ	Unknown	2007 (13)
4	43 M	Dento-alveolar abscess; fever, anemia, diarrhea	Lymphoma; h/o kidney transplant	16s rRNA PCR	None	PCNV + MTZ → PCNV + MTZ; source control: dental extraction	Unknown	2011 (10)
5	66 F	Sepsis; pulmonary abscess	Lung cancer; meningeal carcinomatosis	16s rRNA PCR	None	CXM + GEN → MEM + MTZ + CIP → MTZ	Unknown	2011 (10)
6	64 M	Sepsis	Colon cancer; h/o complicated abdominal surgery	16s rRNA PCR	None	CXM + MTZ; source control: acute exploratory laparotomy (revealed colon cancer relapse)	4 weeks	2011 (10)
7	33 F	Femoral vein thrombosis and abscess	Intravenous drug abuse; chronic hepatitis B	16s rRNA PCR	*Actinomyces meyeri*	CXM → PEN + MTZ	5 weeks	2011 (10)
8	77 M	Pneumonia; toothache	Prostate cancer; chronic heart disease	16s rRNA PCR	*Porphyromonas uenonis*	PCNG → PCNV	4 weeks	2011 (10)
9	56 M	Fournier gangrene	Unknown	Unknown	Unknown	Unknown	Unknown	2017 (17)
10	58 M	Sepsis; bronchiolitis; pyothorax; pulmonary abscess	Lung cancer; COPD; encephalopathy; neuromuscular dysfunction; cachexia	Unknown	None	Declined treatment	Unknown	2016 (12)
11	36 M	Acute cholangitis	Recurrent chronic pancreatitis	16s rRNA PCR and MALDI-TOF MS	None	CTX → MEM → TZP → unspecified maintenance antibiotics	25 days	2020 (14)
12	61 M	Sepsis; thrombotic thrombocytopenic purpura; concomitant brucellosis	Hypertension; hyperlipidemia; type 2 diabetes mellitus; rectal cancer	16s rRNA PCR	*Streptococcus mitis;* sputum culture: ESBL *Klebsiella pneumoniae, Proteus mirabilis*	CXM + MXF → minocycline + rifampicin for brucellosis → VAN + MEM	Unknown	2019 (11)
13	70 M	Sepsis; pneumonia; HSV-1 esophagitis	HIV infection; periodontitis	MALDI-TOF MS	*Campylobacter rectus*	AMC	Unknown	2019 (15)
14	76 M	Sepsis; peritonitis	Colon cancer; diabetes mellitus	16s rRNA PCR	*Bacteroides* *thetaiotaomicron; Escherichia coli*	Unknown	Unknown	2021 (5)
15	85 M	Unknown	Unknown	16s rRNA PCR	*Streptococcus constellatus*	Unknown	Unknown	2021 (5)
16	65 M	Diabetic foot infection	Diabetes mellitus	16s rRNA PCR	*Gemella* spp.	Unknown	Unknown	2021 (5)
17	52 M	Diabetic foot infection	Diabetes mellitus	16s rRNA PCR	*Peptoniphilus asaccharolyticus; Staphylococcus aureus*	Unknown	Unknown	2021 (5)
18	37 M	Peritonsillar phlegmon	Unknown	16s rRNA PCR	*Prevotella* spp.	Unknown	Unknown	2021 (5)
19	93 M	Severe sepsis; diarrhea	Unknown	16s rRNA PCR	None	Unknown	Unknown	2021 (5)
20	42 F	Decompensated alcoholic cirrhosis with ascites	Unknown	16s rRNA PCR	None	Unknown	Unknown	2021 (5)
21	88F	Unknown	Unknown	16s rRNA PCR	None	Unknown	Unknown	2021 (5)
22	70M	Unknown	Unknown	16s rRNA PCR	None	Unknown	Unknown	2021 (5)
23	36M	Unknown	Unknown	16s rRNA PCR	None	Unknown	Unknown	2021 (5)
24	75 F	Appendicular peritonitis; bowel obstruction	Unknown	16s rRNA PCR	None	Unknown	Unknown	2021 (5)
25	19 F	Sepsis; eft maxillary sinusitis; subperiosteal abscess	None	MALDI-TOF MS	None; intraoperative culture: mixed aerobic and anaerobic flora, including *Solobacterium moorei*	AMC + MTZ → AMC; source control: endoscopic sinonasal surgery	18 days	2023 (16)
26	56 M	Sepsis; flexor tenosynovitis	Schizoaffective disorder; polysubstance abuse disorder; ADHD	MALDI-TOF MS	None; intraoperative culture: *Streptococcus constellatus*, *Fusobacterium* spp., *Prevotella* spp.	TZP → SAM → AMC; source control: I&D x3	15 days	2024 (current)

^*a*^ Abbreviations: PCNG—penicillin G; PCNV—penicillin V; PEN—unspecified penicillin; FLU—flucloxacillin; AMC—amoxicillin/clavulanate; SAM—ampicillin/sulbactam; TZP—piperacillin/tazobactam; MEM—meropenem; CXM—cefuroxime; CTX—cefotaxime; FEP—cefepime; VAN—vancomycin; CLI—clindamycin; GEN—gentamycin; MTZ—metronidazole; CIP—ciprofloxacin; MXF—moxifloxacin. The arrows in the treatment column indicate a change in the antibiotic regimen.

*S. moorei* is also associated with dental (4), skin and soft tissue, and wound infections (18). It can cause osteoarticular infections, otitis media, abdominal infections, perirectal abscesses, and even intracranial infections (5, 12, 18). *S. moorei* is usually found in polymicrobial tissue infections with mixed aerobic and anaerobic microorganisms, most commonly *Fusobacterium* species, *Streptococcus constellatus*, *Bacteroides* species, *Actinomyces* species, *Prevotella* species, *Parvimonas micra*, *Staphylococcus aureus*, *Porphyromonas* species, *Escherichia coli*, and *Atopobium* species (5, 9–12, 15, 18). However, in blood cultures, it is typically the only pathogen isolated (Table 1). This aligns with the microbiological findings in our patient. *S. moorei* was not isolated from the wound culture, even though this was the likely source of infection. This is likely due to difficult cultivation and slow growth of *S. moorei* compared to the other pathogens isolated.

Because *S. moorei* exhibits slow growth, has few specific positive biochemical reactions, and displays phenotypic variation, there are no commercially available identification kits (18). MALDI-TOF MS and 16s rRNA polymerase chain reaction (PCR) are the primary diagnostic tools for *S. moorei* identification. Most cases have been identified with 16s rRNA PCR (Table 1), which is considered the gold standard due to slightly higher precision (19). However, MALDI-TOF MS is a reliable alternative with a faster turnaround time and lower cost than 16s rRNA PCR (20). The available models include Bruker Biotyper (Bruker), Vitek MS (bioMérieux), Vitek MS Prime (bioMérieux), EX2600 (Zybio), and MicroIDSys (ASTA) (21, 22). The present case used the Bruker Biotyper Microflex with flexAnalysis 3.4.79.0 software. MALDI-TOF MS requires a pure culture, while 16s rRNA PCR can be done with mixed samples (23). However, this is rarely done routinely due to cost, complexity, and potential decrease in specificity as more microorganisms are identified (24). A laboratory’s ability to identify this microorganism depends on their routine identification procedures. It is easily missed, as it is not included in all identification cards and is not in all MALDI-TOF MS databases.

*S. moorei* bacteremia has been treated with a wide variety of antibiotics (9–11, 13–16). Our review indicates it is reliably susceptible to beta-lactams and clindamycin. Lee et al. reported a case with resistance to both trimethoprim/sulfamethoxazole and rifampin (Table 2) (14). In the present case, E-test using the latest CLSI-M100 standards showed resistance to metronidazole, which contrasts with reports of other blood isolates. A common challenge for anaerobic susceptibility testing is the limited availability of CLSI-approved methods. Other challenges include the need for a stable level of anaerobiosis during incubation, longer turnaround time compared to susceptibility testing for aerobes, the need for extensive staff experience and quality control, and breakpoint differences between CLSI and other used standards (25). Most patients survive *S. moorei* bacteremia, as did the patient described here. There were two reported deaths: one patient with sepsis, bronchiolitis, pulmonary abscess, pyothorax, and lung cancer who declined treatment (12); and another with *S. moorei* bacteremia, pneumonia, and brucellosis who ultimately died of thrombotic thrombocytopenic purpura (11).

**TABLE 2:** Reported susceptibilities in *S. moorei* bacteremia (8–12, 14–16)^*a*^

Antibiotic/antibiotic group	No. of isolates tested (*n*)	Susceptibility (%)
Penicillin	11	100
Aminopenicillins^*b*^	2	100
Aminopenicillins/BLI^*c*^	6	100
Piperacillin/tazobactam	8	100
3rd-generation cephalosporins^*d*^	2	100
Cefepime	1	100
Carbapenems^*e*^	8	100
Vancomycin	9	100
Clindamycin	8	100
Clarithromycin	1	100
Moxifloxacin	5	100
Tigecycline	5	100
Metronidazole	11	91
Rifampin	1	0
Trimethoprim/sulfamethoxazole	1	0
Linezolid	1	0

^*a*^ In the case series by Alauzet et al., antibiotic susceptibilities were tested on 24 of 27 cases, with all being susceptible to beta lactams and metronidazole and 4.3% of tested isolates being resistant to clindamycin, 11.8% to moxifloxacin, and 91.3% to rifampin. Since this study did not specify which findings were in the cases of bacteremia, these data were not included in the table (4).

^*b*^ Amoxicillin, ampicillin.

^*c*^ Amoxicillin/clavulanate, ampicillin/sulbactam.

^*d*^ Ceftriaxone, cefotaxime.

^*e*^ Meropenem, imipenem.

There are no specific guidelines for the management of anaerobic bacteremia, specifically for treatment duration and whether echocardiogram is necessary. Previous cases reported a treatment duration of 2 to 5 weeks (Table 1). In the present case, the patient was treated for 15 days from the final I&D.

### Conclusion

*S. moorei* sepsis with bacteremia is a very rare infection, with only 26 cases (including this case) reported. It is usually associated with underlying diseases, such as malignancy, often with compromised integrity of protective skin and mucosal barriers. The literature review indicates isolates are typically susceptible to penicillins, beta-lactam/beta-lactamase inhibitors, carbapenems, and 3rd/4th-generation cephalosporins but may be resistant to metronidazole, levofloxacin, and rifampin. Improvements in diagnostic methods and expansion of databases, such as for MALDI-TOF MS, may yield more frequent diagnosis of this infection and distinguish it from other types of anaerobic bacteremia.
